# Improving Program Outcomes Through Responsive Feedback: A Case Study of a Leadership Development Academy in Nigeria

**DOI:** 10.9745/GHSP-D-22-00121

**Published:** 2023-12-18

**Authors:** Teslim Aminu, Onajite Otokpen, Ijeoma Mmirikwe, Oluwasegun Adetunde, Ibidun Ajuwon, Adesina Adelakun, Abdulateef Salisu, Faisal Shuaib, Uchenna Igbokwe, Muyi Aina

**Affiliations:** a Solina Centre for International Development and Research, Abuja, Nigeria.; b National Primary Health Care Development Agency, Abuja, Nigeria.

## Abstract

**Background:**

In 2018, the National Primary Health Care Development Agency (NPHCDA), under the Nigeria Federal Ministry of Health, recognized that inadequate personnel competencies limited its ability to provide technical assistance to state primary health care development boards (SPHCBs). In response, NPHCDA instituted a cohort-based leadership development academy (LDA) to develop its staff's leadership and management skills.

**NPHCDA Leadership Development Academy Program:**

A baseline assessment identified critical gaps in leadership and management competencies. Agency leaders and midlevel staff aligned on the gaps and approaches to bridge them. Participants in the LDA were selected across headquarters and zones to ensure equitable spread. The NPHCDA adopted a mix of capacity-building approaches that prioritized 6 core leadership and management skills with the application of the 70–20–10 learning philosophy to ensure participants learn and apply skills. Responsive feedback (RF) mechanisms were key in program design and implementation. NPHCDA collaborated with development partners through an iterative cocreation process to design and revise for implementation. The program embraced information and communication technology to collate qualitative and quantitative data across activities in real-time and used insights to drive continuous improvement.

**Results:**

Incorporating feedback from the first cohort led to an increase in the proportion of trainees acquiring intermediate to advanced competencies in target skill areas from 41% to 57% in the second cohort. The trainees have taken on more challenging roles at NPHCDA, delivering technical assistance to SPHCBs and bolstering the agency's ability to independently execute activities such as the COVID-19 vaccine introduction. The agency continues to adopt RF mechanisms in the coordination and delivery of advanced technical training for staff and institution of a capacity-building and acculturation program for new hires.

**Conclusion:**

RF has demonstrated that identifying and engaging key stakeholders to participate in continuous learning and adaptation is critical to achieving program effectiveness and sustainability.

## Abstract (precis)

The National Primary Health Care Development Agency in Nigeria used a responsive feedback mechanism to successfully establish a leadership development academy for building core leadership, management, and basic functional skills among its staff.

## Teaser

**Key Findings:**

- The National Primary Health Care Development Agency (NPHCDA) established a leadership development academy (LDA) to build core leadership, management, and basic functional skills in cohorts of 25–30 NPHCDA staff over 6-month cycles to coordinate and provide technical assistance interventions to all state primary health care boards.
- Capacity-building can lead to skill development if well aligned with clearly defined organizational functions and based on clear theories of change and action that must be developed together with the critical stakeholders within the organization.
- Program design must be suited to change and adaptation based on evidence generated from the key stakeholders involved, and changes driven by evidence from program data analysis can potentially increase the desired outcomes.

**Key Implications:**

- Public-sector organizations have the opportunity to incorporate responsive feedback (RF) in the design and implementation of capacity-building interventions to ensure the desired outcomes are achieved with mutual ownership.
- NPHCDA should leverage the learnings from the adoption of RF mechanisms in its LDA program design and implementation to improve other program outcomes.

## BACKGROUND

Many public-sector organizations are challenged by their employees' lack of the required leadership and management skills to contribute to organizational performance.^1^ Such a skill gap can be the result of expanding agency roles, complexity of the work environment, or rapid changes in organizational and technological advancement within the agency.

Equipping their workforce with the required skills has now become a strategic concern for most employers, thereby making training a necessity for public-sector workers. Organizations often hold staff capacity-building sessions to bridge the capacity gaps. Although some of the capacity-building approaches deployed across public-sector organizations have shown a positive impact on the trainees and organizations, not all of them have led to the expected outcomes.^2^ In 2015, U.S. companies were reported to have spent US$356 billion globally on employee training and education without getting a good return on investment, as the learnings did not lead to better organizational performance because people quickly reverted to their former way of doing things.^3^

Equipping their workforce with the required leadership and management skills has now become a strategic concern for most employers.

Failure of training efforts is due to factors such as lack of buy-in of the organization's leadership; use of capacity-building interventions that are not tailored to address identified gaps; an inappropriate mix of training approaches; and lack of effective monitoring, evaluation, and learning systems to measure skill gain and improve the learning approach to yield the expected outcomes. Previous reviews have reported the adoption of mainstream approaches not contextualized to the realities within the organizations as a significant barrier to translating capacity interventions to organizational performance.^4^ It was also opined that most training for employees is never properly contextualized or focused on the individual's needs using structured approaches, which means it does not ultimately contribute to organizational growth.^5^ However, it has been argued that capacity-building might not be as effective because the approaches used might be inadequate.^6^ In another study, it was suggested that capacity-building is more effective if the target organization is prepared to receive the support.^7^ When a significant amount of time and money is spent on training without taking individual training needs into account, such exercises are based on erroneous diagnostics and are often unsuitable to address the capacity needs.

Responsive feedback (RF) has become a core concept in program design and execution in the development industry in recent years. An RF mechanism is a system that ensures all the stakeholders relevant to a project are equitably engaged in the design and implementation of the project. It helps to promote interaction between project designers, implementers, researchers, and decision-makers to encourage adaptation.^8^ This helped the NPHCDA course correct and iterate as the program is designed and implemented to ensure the effectiveness of the program. An RF mechanism provides room for flexibility based on feedback from the end-users, beneficiaries, and other strategic stakeholders. This feedback is continuously collated and reviewed during appropriate pause-and-reflect sessions to improve the design, quality of delivery, project performance, and impact, which becomes more targeted and relevant to the beneficiaries.

An RF mechanism ensures that all the stakeholders relevant to a project are equitably engaged in the design and implementation of the project.

Incorporating RF into the design, implementation, and learning of capacity-building programs is key to ensuring their successful outcomes, which in turn drives organizational transformation. Limited flexibility in programmatic response is often seen when little or no feedback is provided to decision-makers, including implementers, during the implementation process. Past studies identified the lack of design iteration as a challenge encountered in designing and implementing capacity-building interventions.^9^ Further reviews also buttressed the fact that the culture of an organization can influence the execution of an RF mechanism.^8^ This lack of agility may be a function of the types of data, study designs, and management skills needed to respond to changing implementation needs. It is equally possible that an organization's culture may not be open to receiving and acting on feedback.

In 2018, the leadership of the National Primary Health Care Development Agency (NPHCDA), along with its funder, the Bill & Melinda Gates Foundation, and the implementing partner, Solina Centre for International Development and Research, recognized the importance of RF and used it as a guiding principle to establish a long-term capacity-building program to improve the leadership and management capabilities of its staff. A major success factor in the implementation of the capacity-building program was the continual improvement in the program design and implementation through feedback from all stakeholders.

## NPHCDA LEADERSHIP DEVELOPMENT ACADEMY PROGRAM

### Program Justification

Our case study documents how an RF mechanism was used to establish an effective and sustainable capacity-building program to address identified gaps in leadership and management skills at the NPHCDA. The NPHCDA recognized the lack of sufficient staff with management competencies needed for the effective delivery of transformational primary health care (PHC) initiatives. To bridge this gap, the agency had previously deployed a few capacity-building interventions that had not yielded favorable results. In line with the drive to fulfill the agency's mandate, the Executive Director, Dr. Faisal Shuaib, envisioned a leadership development academy (LDA) as a means to bridge the identified capacity gaps. An appropriate project management approach for developing a capacity development initiative within the agency was needed. The decision was made to adopt an RF mechanism to allow for collaborative development of the LDA and to ensure effective coordination and program quality.

### NPHCDA Capacity-Building Framework

**FIGURE 1:**
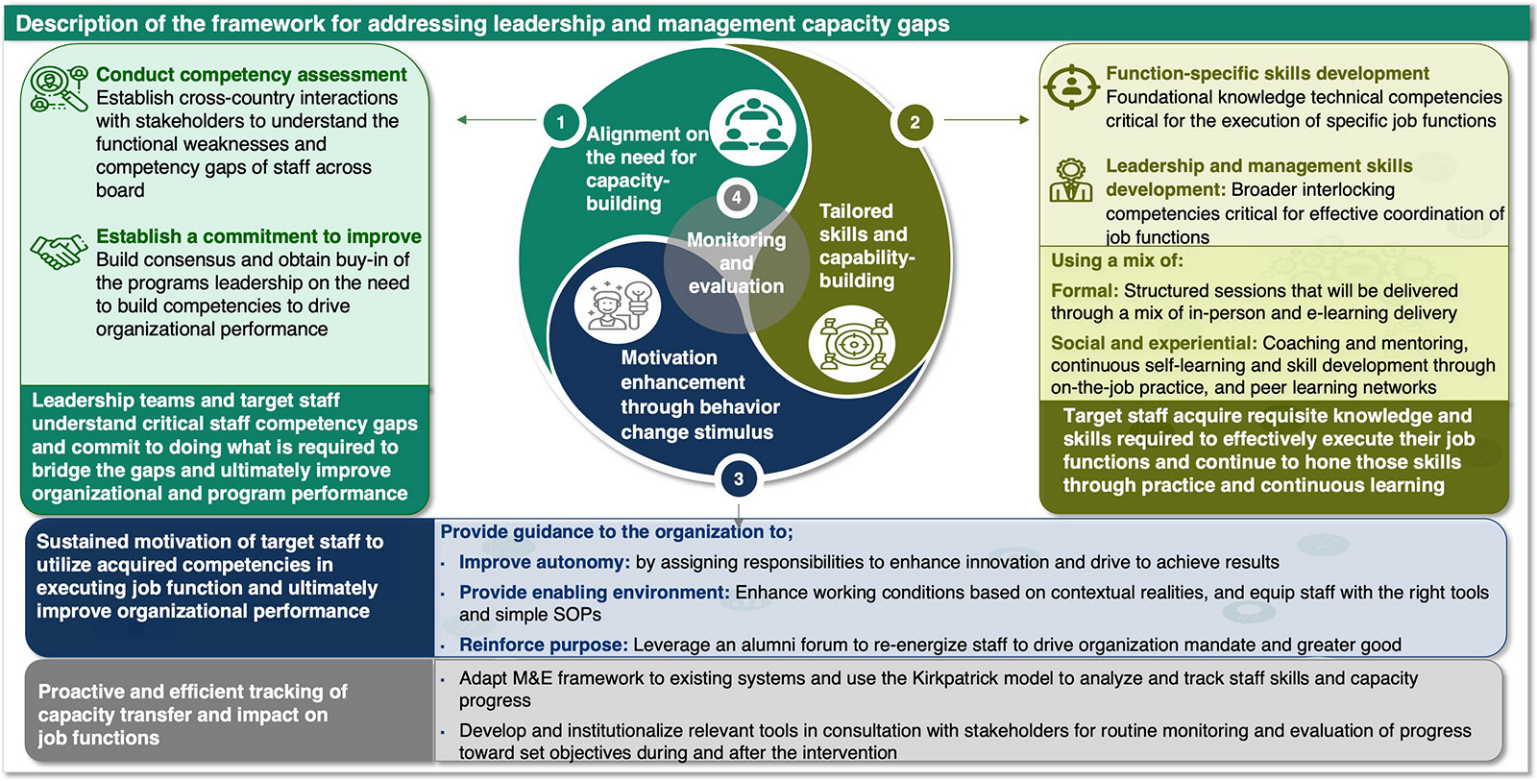
NPHCDA Capacity-Building Framework^a^ Abbreviations: M&E, monitoring and evaluation; NPHCDA, National Primary Health Care Development Agency; SOP, standard operating procedure. ^a^ Source: Solina Centre for International Development and Research.

The NPHCDA employed a multistakeholder approach over several iterative sessions to develop a framework for building and sustaining staff capacity. The capacity-building framework was developed using RF approaches, taking into account feedback from the design stage to program execution, before arriving at the final structure shown in Figure 1. The framework is categorized into 4 themes: alignment on the need for capacity development, tailored skills and capacity-building motivation enhancement through behavioral change stimulus, and monitoring and evaluation (M&E).

The NPHCDA employed a multistakeholder approach over several iterative sessions to develop a framework for building and sustaining staff capacity.

#### Alignment on the Need for Capacity-Building

The senior NPHCDA leaders aligned on the need for capacity-building to improve functional weaknesses. To identify the focus of the LDA program, senior NPHCDA leaders used a customized system and program diagnostics tool to identify the critically underperforming functions/business units impeding achievement of the organization's mandate. The results showed a lack of technically capable staff to effectively lead and coordinate technical assistance (TA) delivery to the state primary health care development boards (SPHCBs), which in turn hampered the agency's ability to achieve its mandate of providing TA and programmatic leadership for PHC service delivery in Nigeria. The agency constituted a steering committee drawn from departments of the agency to (1) oversee the process for developing staff capacity to bridge the identified gaps and (2) ensure frequent engagements with the leadership through the process. A theory of change was developed to identify the critical functions necessary for the program to achieve the desired result and articulate the underlying assumptions in need of validation. Critical leadership and management competency gaps were identified through a needs assessment. This was followed by alignment between agency executives and midlevel staff on approaches to address the gaps and on their commitment to the achievement of program goals.

#### Tailored Skills and Capacity-Building

**FIGURE 2:**
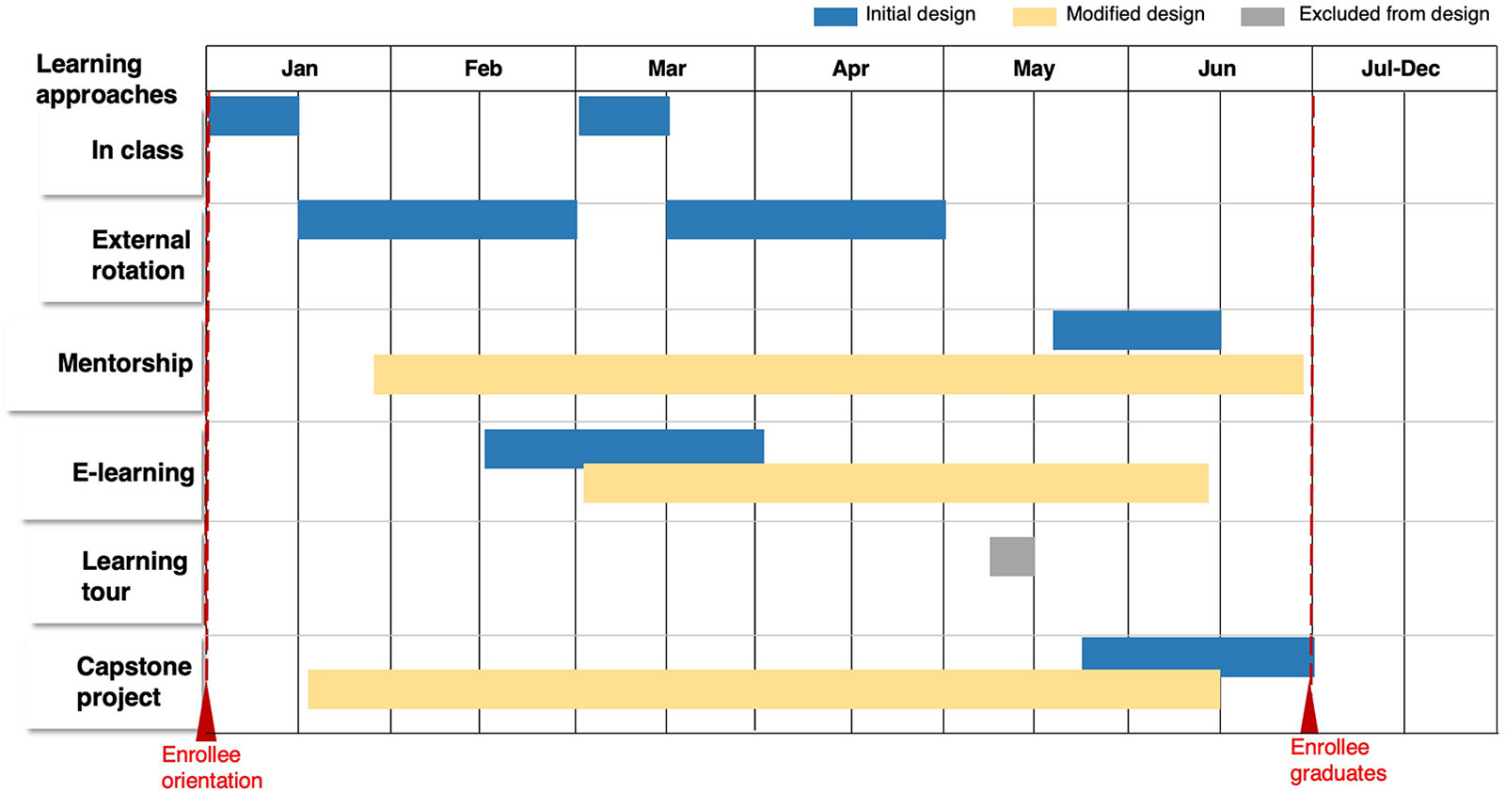
Modifications to the Leadership Development Academy Program Implementation Timeline Based on Received Feedback^a^ ^a^ Source: Solina Centre for International Development and Research.

To design the capacity-building program (Table 1) and ensure participants learn and apply requisite skills (Figure 1), the NPHCDA adopted the 70–20–10 learning philosophy (70% experiential: continuous self-learning and skill development through on-the-job practice; 20% social: coaching and mentoring, job shadowing, external rotations, and peer learning networks; 10% formal: structured in-class and e-learning courses). The NPHCDA held a series of brainstorming sessions with its technical partners—including the Solina Centre for International Development and Research, World Health Organization, Clinton Health Access Initiative, and Africa Resource Centre for Supply Chain—to identify the most efficient capacity-building approaches based on international best practices to optimize staff learning outcomes. This was accomplished through a variety of adult learning methods. All the learning approaches were provided concurrently, with the exception of the capstone project and e-learning, which were carried out in parallel (Figure 2). Based on the results of the needs assessment and internationally recognized best practices, the curriculum prioritized 6 fundamental leadership and management skills, including effective communication, problem-solving, team management, stakeholder management, programmatic, and core ethics (Table 2). Subject matter experts from partner organizations and the private sector were engaged to develop, review, and deliver the training modules as well as supervise trainees' skill development.

**TABLE 1.** Responsive Feedback Mechanism Stepwise Design for NPHCDA Leadership Development Academy

Focus and Description	Data Collected, Data Collection Tools, and Period of Collection	Stakeholders Involved	Meeting Fora to Discuss Data Findings and Objective Frequency
**Program design**			
Align on: Capacity-building support Defining the scope of capacity-building Plan to deploy the LDA	Baseline assessment result; Baseline assessment forms; 3 months	Executive director, departmental directors, TSU, supporting partners	Kick-off meeting and then held weekly until program commencement To align on the need for capacity development and plan to build and sustain staff capacity
**RF incorporation:** Held multiple iterative sessions with the leadership of the NPHCDA and partner organizations to elicit feedback on the capacity-building design before rollout. Feedback from key stakeholders was reflected in the design by using baseline assessment results and interviews to screen and select enrollees to ensure commitment as opposed to the use of the civil service quota system. Initial program design allocated to span for 12 months but reduced to 6 months based on feedback from the NPCHDA leadership to ensure that staff do not lose focus of their assigned job functions at the agency.			
**Implementation (by learning approaches)**			
In-class session: Ensure trainees acquire the requisite knowledge to develop the focus skills of the LDA Interactive lectures Demonstrations Individual and group exercises	Pre- and post-assessment results; Baseline assessment evaluation form, in-class session assessment form, course delivery feedback form; 4 weeks (in 2 waves of 2 weeks each, where the second wave commences after the first wave of external rotation)	TSU, steering committee, enrollees	Program review meetings Held once To review assessment results, identification of trainees' strengths and weaknesses to inform area of support from the other learning approaches
External rotation: Provide firsthand experience of different work cultures and the opportunity to apply knowledge gained and develop skills	Logbook, supervisor's ratings; Logbook, performance appraisal, check-in visit feedback form; 12 weeks (in 2 waves of 6 weeks each that commences after each in-class session)	TSU, externship supervisors from partner organizations	Program review meetings held bi-weekly To review trainee's performance
Mentorship: One-on-one sessions with an experienced professional to support the trainees to own their personal and professional leadership growth and development	Midpoint evaluation tracker, mentee feedback form, mentor feedback form; 20 weeks (runs concurently with the external rotation and e-learning)	TSU, mentors, steering committee	Program review meetings held monthly To review trainee's activities towards achieving a personal development plan. To review implementation of mentor-prescribed activities
E-learning: Expose trainee to carefully curated PHC content across multiple learning platforms to facilitate continuous learning	Certificates of course completion Feedback forms, weekly check-in calls tracker; 10 weeks (runs concurrently with the external rotation)	TSU, steering committee	Program review meetings held weekly To review the status of completion of e-courses
Capstone project: Apply all the knowledge and exhibit skills gained from the other learning approaches to plan and deliver technical assistance requests from SPHCBs	Performance evaluation form; 13 weeks (commences after the first wave of external rotation and runs until the end of the LDA)	TSU, partners, steering committee, secretariats, division directors, SPHCB	Program review meetings held biweekly To review implementation of capstone project; challenges and lessons learned
Learning tour: Expose enrollees to successfully executed interventions outside Nigeria through discussions, site visits, and interviews along the health building blocks that are directly applicable to Nigeria's PHC priorities	Learning tour report; 1 week	Executive director, directors of divisions, TSU, Ministry of Health representatives, steering committee, supporting partners, other country's program directors	Senior management meetings and program review meetings Held once To review program design, implementation, and results
**RF incorporation:** Incorporated feedback from the enrollees, supervisors, and steering committee on the need for an objective evaluation of the external rotation. Introduced a logbook and performance appraisal forms after the first wave of the first cohort. Reviewed enrollees' feedback forms that revealed that some partner organizations did not offer an enabling environment (workspace and work assignments) for the enrollees to enable them to accomplish their milestones. As a result, postings were made based on the partner organization's ability and willingness to do so. Observed a mismatch of enrollees to partner organizations based on the skills they need to acquire and made appropriate changes after receiving feedback from the enrollees and alignment with the steering committee. To sustain learning enthusiasm, integrated cocurricular activities, such as picnics and weekend aerobic sessions, in the second wave of the in-class session based on enrollees' feedback.			
**Review**			
End of cohort review: Assess LDA enrollees on their view of the learning approaches for course correction and improvement	LDA assessment result, LDA report; LDA assessment form, post-LDA assessment form; 4 weeks	Executive director, division directors, TSU, steering committee, supporting partners	Senior management meetings, program review meetings Held once To review program design, implementation, and results
**RF incorporation:** Made modifications to the curriculum during the first cohort review. Deprioritized vision and strategic thinking courses based on enrollees' feedback that the skill could not be applied within the agency. Introduced a time management course to increase enrollees' work productivity. These changes were reviewed and approved by the steering committee. Removed learning tour from the learning strategy because it contributed very little to the skill or knowledge growth of the enrollees.			

Abbreviations: LDA, leadership development academy; NPHCDA, National Primary Health Care Development Agency; PHC, primary health care; SPHCB, state primary health care board; TSU, technical support unit.

**TABLE 2.** Fundamental Leadership and Management Skills Prioritized by Leadership Development Academy Curriculum

Skill	Focus Skills
Effective communication	1. Written communication
	2. Public speaking
Problem-solving	3. Innovation and creativity
	4. Analytical reasoning and critical thinking
Team management	5. Work planning and budgeting
	6. Influencing and negotiation
	7. Staff performance management
	8. Conflict management
	9. Team work
	10. Coaching and feedback
	11. Time management strategies
	12. Vision and strategic thinking^a^
Stakeholder management	13. Stakeholder management
	14. Meeting and workshop management
Programmatic	15. Monitoring and evaluation
	16. Introduction to research
	17. Continuous learning and personal development
Core ethics	18. Institutional and personal ethics

a Excluded in cohort 2 based on feedback.

Source: Solina Centre for International Development and Research.

**FIGURE 3:**
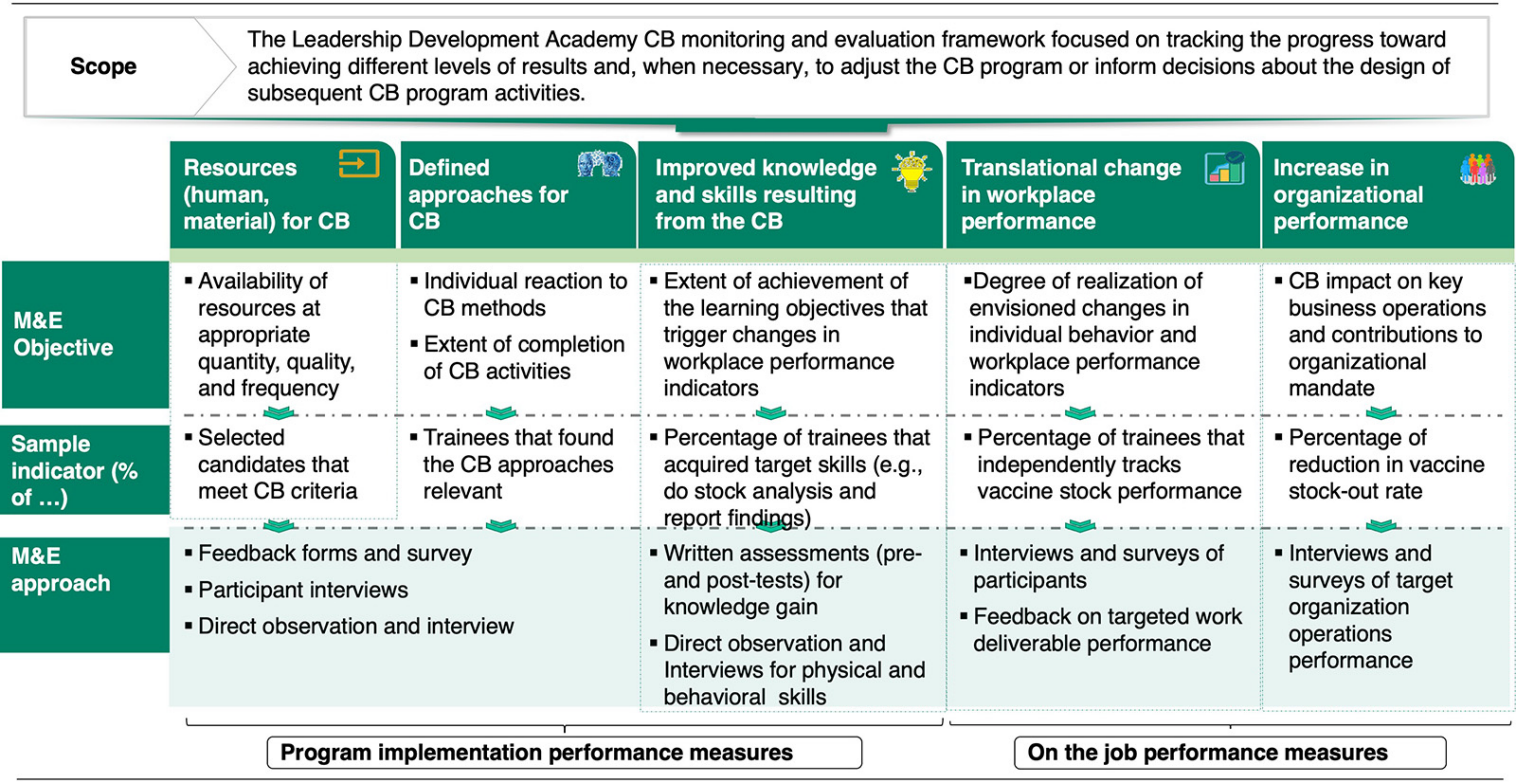
M&E Framework for NPHCDA Leadership Development Academy^a^ Abbreviations: CB, capacity-building; M&E, monitoring and evaluation; NPHCDA, National Primary Health Care Development Agency. ^a^ Source: Solina Centre for International Development and Research.

A robust monitoring M&E system (Figure 3) was designed for the program, embracing information and communication technology to rapidly collate real-time data across the program activities, allowing for informed adjustments during implementation and driving continuous improvement. The data included pre-test and post-test scores for the class sessions to measure knowledge gain; quality of facilitation of the facilitators; performance level of the enrollees as they perform tasks at the partner organizations; number of certificates acquired from the e-learning courses; and skill growth level as they execute their capstone project (Figure 3). The agency collaborated with development partners and institutions through an iterative process of continuous cocreation to design the program curriculum. The team routinely collated data on the most important learning concepts for the in-class session modules and modified them to include key concepts or exclude those that were deemed irrelevant through reviews and feedback forms. The program collected and analyzed the critical gaps identified from the baseline assessment and obtained feedback from the facilitators of each course module.

A robust M&E system was designed to allow for informed adjustments during implementation and drive continuous improvement.

#### Motivation Enhancement Through Behavior Change Stimulus

The NPHCDA sought to ensure trainees had deliberate opportunities for on-the-job skill application upon completion of the LDA program to drive job performance by improving autonomy and providing an enabling environment to function effectively (Figure 1). Some staff were appointed as unit leads, and others were assigned new job functions and additional tasks in line with their competency development. Additionally, staff were provided with requisite tools to aid optimal performance and help them serve as change agents within the unit and department by coaching the NPHCDA's younger employees. Following program completion, NPHCDA senior management was tasked with determining the best method to support the enrollees through participation in senior leadership activities, including stakeholders' engagement meetings and specific organizational growth activities to improve ownership.

#### Monitoring and Evaluation

Solina Centre for International Development and Research adopted an M&E framework based on the Kirkpatrick model—which uses 4 levels of evaluation to measure reaction, learning, behavior, and results^10^—to analyze and track skill gain and progress made by staff during and after the capacity-building intervention. This ensures that the objectives and targets of the program are achieved. The information and skills acquired during the application of the learning methodologies were monitored and measured to further identify areas for development and to record lessons learned to direct subsequent interventions.

### RF Approach in LDA Design

As shown in Table 1, several levels of data were collected from different stakeholders at various points to help inform the evidence-based decisions in program design. The agency engaged relevant stakeholders, including senior management staff, the technical support unit, and partner organizations, to align and conduct a capacity needs assessment of its staff. The steering committee was also constituted to oversee the capacity interventions for the LDA. The assessment results and the validation sessions with stakeholders informed the decisions on the key concepts to be taught and learning approaches required to bridge identified gaps. RF was key in the LDA program design and implementation; the agency collaborated with developmental partners and institutions through an iterative process of continuous cocreation to design and periodically revise for implementation. Across all of the learning approaches deployed, the LDA used feedback forms and interviews to elicit feedback from enrollees to improve overall program outcomes. RF was adopted by engaging the agency's leadership to design the curriculum to effectively address the gaps identified from the needs assessment and by working with the steering committee to increase the number of check-ins with the enrollees so that the requisite support and time allotted for each course were sufficient. Following the implementation of the first cohort, RF was also employed to improve the implementation of the second cohort. Kirkpatrick's model was used by the technical support unit and LDA steering committee to evaluate the capacity-building program's performance. The team closely monitored all aspects of the program to ensure optimal quality of each learning approach. The team customized various assessment tools and instituted weekly check-in sessions to ensure trainees' participation in the program and resolve all challenges as they arise. The enrollees' proficiency levels were evaluated at the beginning and the end of the LDA program to measure growth and ascertain impact of the interventions.

RF was key in the LDA program design and implementation.

## RESULTS

The incorporation of RF at every stage of the NPHCDA capacity-building program yielded positive results. The results are discussed across 3 themes: theory of change and design modification, capacity-building results, and post-implementation impact reflecting changes that were informed from RF between the first and second cohorts.

### Theory of Change and Design Modification

At the end of the first cohort LDA program review, we sought to identify key areas of the program design that required innovative changes to optimize the desired program outcome for the enrollees. The team engaged the steering committee and donor in an internal brainstorming meeting to review critical areas of implementation along the learning approaches, identify what had worked well and what did not, and determine the opportunities for improvement. One of the key decisions reached was on how to incorporate a suitable timeline for the completion of 3 learning approaches. An overwhelming number of enrollees had requested more time to complete the mentorship, e-learning, and capstone project. The initial design, which comprised concurrent delivery of the learning approaches, did not allow enrollees to optimize time effectively across the interventions. To respond to the feedback, as shown in Figure 3, the technical support unit and steering committee agreed to redesign the timeline for the implementation to allow for overlaps between learning approaches, with an extra 3 months provided for completion of the learning interventions.

In response to enrollee feedback, the timeline was redesigned to allow for overlaps between learning approaches and extra time for completion.

In response to the analysis, reviews, and feedback from the key stakeholders, changes were made to key components of the learning interventions design.

#### In-Class Session

For the in-class session, we deprioritized the vision and strategic thinking course module and replaced it with time management strategy, included a reflection session after the completion of the in-class session, and revised the pre- and post-assessment from objective-type questions to open-ended case studies to effectively test understanding of key concepts taught.

#### External Rotation

Organizations for the external rotation were chosen based on their history of working with the NPHCDA to support PHC initiatives and their dedication to giving enrollees the chance to develop soft leadership skills, like teamwork and problem-solving abilities, as well as technical PHC skills across the fundamental PHC building blocks. Some organizations were dropped during the second cohort's implementation because the enrollees couldn't apply and internalize these abilities there. The criteria for selecting organizations to support the external rotation was expanded to ensure fit and capability to support enrollees' milestone development based on feedback from trainees and supervisors during on-site supervisory visits to the trainees at their externship organizations.

#### E-Learning

The number of recommended PHC e-learning courses that targeted knowledge gain in line with enrollees' job functions was increased by over 100% (with 4 new courses added) based on feedback about the effectiveness and popularity of the course modules among enrollees (as they found them applicable to current job roles).

#### Mentorship

Feedback on the rigidity of the mentor-mentee selection process in the first cohort led to the modification of the selection process in the second cohort to allow mentors to select mentees from the pool of enrollees. As a result, mentors felt a lot more responsible for their mentees and supported them in refining their professional development plans. Remarks from the enrollees underline the effectiveness of the mentorship approach.

*My mentor is very nice, accommodating, and thorough. He left no stone unturned. He taught me to write my vision statement, align my core values with the NPHCDA mission and develop a Professional Development Plan (PDP). He even agreed to continue the mentorship after the officially stipulated period. I have a confidence boost.*—Enrollee

*He (my mentor) taught me to aim for the stars. He instilled a belief in me that I should aim as high as possible when setting goals. The experience has also strengthened my resolve to be a humble individual. Seeing how my mentor is a very busy and high-ranking official treats me with respect and level-headedness.* —Enrollee

#### Learning Tour

The learning tour intervention was deprioritized due to limited evidence of its contribution to overall staff capacity development in the LDA.

### Adaptations to COVID-19 Restrictions

The COVID-19 pandemic also ushered in new challenges for program implementation. Key government restrictions and social-distancing measures meant the program activities had to be paused indefinitely between March and June 2020. To ensure the program commenced and still achieved the desired outcomes, the team incorporated feedback to improve the program design along 2 major decision points.

We conducted virtual sessions for capacity-building activities with minimal implication for the overall LDA objectives. The in-class session, external rotation, and capstone projects were all deployed virtually as a result. Based on data from literature reviews and feedback from the steering committee, we made specific changes to these interventions. We adapted the curriculum to include courses on how to “improve remote working productivity” and “manage virtual meetings.” We supported enrollees to commence external rotation virtually with partner organization (or in line with the working modalities of the host organization) in close liaison with their assigned supervisors while assigning a member of the steering committee to each enrollee. We adapted the capstone projects to allow remote TA supports in line with SPHCB needs in response to the COVID-19 pandemic.

We provided virtual facilities necessary to ensure the effectiveness of each learning approach deployed. Specific changes included providing logistics funds to the enrollees for purchase of modems, internet data, and electricity units to ensure all resources needed for successful in-class sessions; and sharing guides on how to install and navigate the virtual conferencing platform software with all participants (focus on Zoom Application)

### Capacity-Building Results: Comparison of the First and Second LDA Cohorts

The LDA has graduated 2 cohorts of 29 and 27 fellows between 2019 and 2020. Incorporation of feedback from the first cohort led to an increase in the proportion of trainees acquiring intermediate to advanced competencies in target skill areas from 41% in the first cohort to 57% in the second cohort.

Incorporating feedback from the first cohort led to an increase in the proportion of trainees acquiring intermediate to advanced competencies in target skill areas in the second cohort.

**FIGURE 4:**
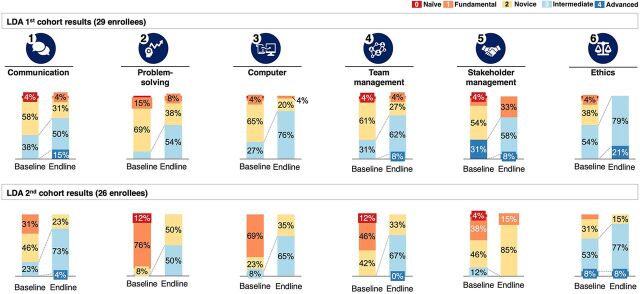
Comparison of Performance Across Defined Competencies of Participants in Cohorts 1 and 2 of the Leadership Development Academy^a^ Abbreviation: LDA, leadership development academy. ^a^ Source: Solina Centre for International Development and Research.

Both the baseline and endline performance of the enrollees were measured using a 5-point rating scale (Box). The endline performance was measured using the capstone project execution. At the end of the training, 73% of the first cohort enrollees and 88% of the second cohort enrollees graduated with skill levels of 3 and above. A comparison of the outcomes between the first and second cohorts of enrollees is shown in Figure 4. The fact that staff members were chosen from the same pool and high performers were chosen each time while others were given recommendations for self-development in advance of the upcoming cohort selection can explain the lower baseline scores seen in the second cohort.

**Figure:**
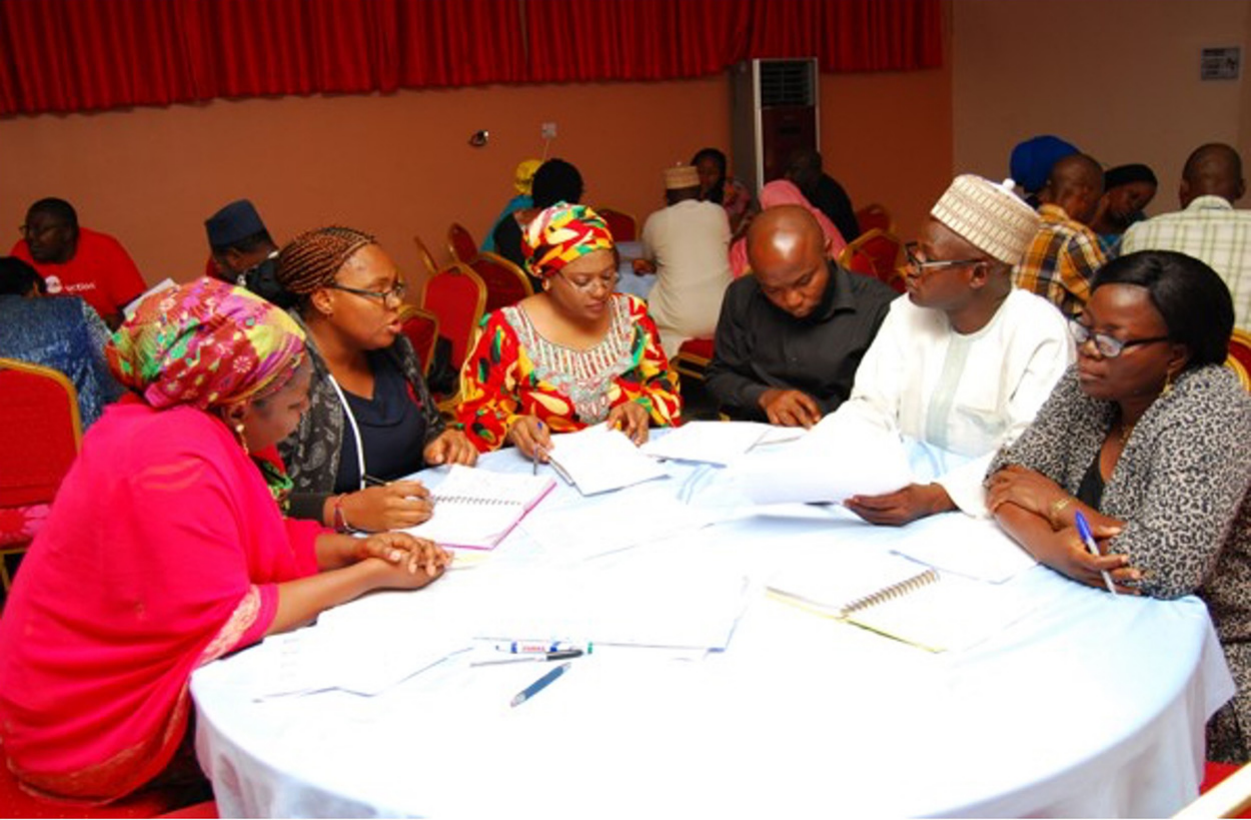
Leadership Development Academy enrollees participate in an in-class session. ©2020 Victor Odiba/Solina Centre for International Development and Research

BOX Performance Rating Scale for Leadership Development Academy Enrollees

0–Naive (no understanding of the skill)

1–Fundamental (has understanding of basic techniques and concepts but requires a lot of support)

2–Novice (can carry out tasks with limited experimental scenarios but will need help when performing the skills)

3–Intermediate (can successfully execute skill independently but might require help from an expert from time to time)

4–Advanced (executes skill without assistance and a go-to person when difficult questions arise regarding the skill)

### Post-Implementation Outcomes and Feedback

A total of 91 TA support requests that cut across the PHC building blocks (e.g., development of annual operational plans and minimum service packages) have so far been fulfilled across 28 SPHCBs in all 6 geopolitical zones of the country through several approaches in line with the program design and responsive to the states' needs. The new trainees have taken on more challenging roles at NPHCDA that include the delivery of TA to SPHCBs and support of the agency's ability to independently execute activities such as the 2020 integrated medical outreach program and the COVID-19 vaccine introduction. The agency has also adapted the LDA to coordinate and deliver advanced technical skills training for staff and instituted a capacity-building and acculturation program for new hires.

The new trainees have taken on more challenging roles at NPHCDA that include the delivery of TA to SPHCBs and support of the COVID-19 vaccine introduction.

Prior to the institution of the LDA, most midlevel staff lacked the requisite skills to deliver TA to states, relying largely on partner support to plan and deliver TA. This limited the amount and quality of support available to SPHCBs. As a result of the training, the fellows have bolstered the agency's ability to execute activities that typically would have required external consultants between 2019 and 2020, saving considerable financial resources for the agency and its partners.

The training has also further improved the work culture and competency of the staff to execute their job functions independently while taking on additional responsibilities.

*The LDA training was really impactful for me, it really boosted my confidence and ability to make fast decisions. This training has helped me understand benefits of differences in opinion irrespective of who has them, or what they are.* —First cohort LDA fellow

*The LDA program was a wonderful turnaround experience for me as it increased my capacity in delivery of all assigned tasks in the office. The LDA has also given me a better approach to dealing with daily activities.* —First cohort LDA fellow

The LDA fellows have gone on to independently plan and deliver TA to SPHCBs, helping to drive PHC transformation in a bid to achieve universal health coverage. Most importantly, they are championing COVID-19 vaccine delivery and distribution by ensuring availability of vaccines in all states and seamless execution of the vaccination process. One fellow, who has been appointed to head the information and communication technology unit of the agency, led a team that designed and implemented the COVID-19 vaccination process from client registration to tracking of dose uptake. Another fellow now independently coordinates and implements the LDA at the agency. The skills these fellows acquired from the LDA have been instrumental to these achievements.

## CONCLUSION

The NPHCDA LDA implementation has demonstrated that an RF mechanism can be applied to capacity-building interventions at public institutions within low- and middle-income country settings. There are many more areas for potential application of this management approach within the NPHCDA and the public sector at large. The post-implementation impact has shown the need for sustained application of this model to improve staff technical capacity, thus creating a critical mass of highly skilled staff across all cadres of management within the organization. The NPHCDA has institutionalized the LDA by creating a separate budget line for it, establishing a technical support unit as a coordinating unit, designating a pool of resource persons for faculty, creating knowledge products, and defining systems and processes for program execution. The agency's senior management should ensure continued funding of the LDA while iteratively harvesting and implementing RF across all relevant stakeholders. To date, the NPHCDA has graduated 2 LDA cohorts with a plan in place for its third cohort, while adopting RF as the bedrock for program modification and execution.

## Notes

**Peer Reviewed**

**First published online:** May 16, 2023.
